# *Clostridioides difficile* colonization is not mediated by bile salts and utilizes Stickland fermentation of proline in an *in vitro* model

**DOI:** 10.1128/msphere.01049-24

**Published:** 2025-01-16

**Authors:** Xiaoyun Huang, April E. Johnson, Joshua N. Brehm, Thi Van Thanh Do, Thomas A. Auchtung, Hugh C. McCullough, Armando I. Lerma, Sigmund J. Haidacher, Kathleen M. Hoch, Thomas D. Horvath, Joseph A. Sorg, Anthony M. Haag, Jennifer M. Auchtung

**Affiliations:** 1 Department of Food Science and Technology and Nebraska Food for Health Center, University of Nebraska-Lincoln, Lincoln, Nebraska, USA; 2 Department of Biology, Texas A&M University, College Station, Texas, USA; 3 Department of Pathology and Immunology, Baylor College of Medicine, Houston, Texas, USA; 4 Department of Pathology, Texas Children’s Microbiome Center, Texas Children’s Hospital, Houston, Texas, USA; 5 Department of Pharmacy Practice & Translational Research, University of Houston, Houston, Texas, USA; The University of Iowa, Iowa City, Iowa, USA

**Keywords:** Stickland fermentation, bile metabolism, microbiota, antibiotic disruption

## Abstract

Treatment with antibiotics is a major risk factor for *Clostridioides difficile* infection, likely due to depletion of the gastrointestinal microbiota. Two microbiota-mediated mechanisms thought to limit *C. difficile* colonization include the conversion of conjugated primary bile salts into secondary bile salts toxic to *C. difficile* growth and competition between the microbiota and *C. difficile* for limiting nutrients. Using a continuous flow model that simulates the nutrient conditions of the distal colon, we investigated how treatment with 6 clinically used antibiotics influenced susceptibility to *C. difficile* infection in 12 different microbial communities cultivated from healthy individuals. Antibiotic treatment reduced microbial richness; disruption varied by antibiotic class and microbiota composition, but did not correlate with *C. difficile* susceptibility. Antibiotic treatment also disrupted microbial bile salt metabolism, increasing levels of the primary bile salt, cholate. However, changes in bile salt did not correlate with increased *C. difficile* susceptibility. Furthermore, bile salts were not required to inhibit *C. difficile* colonization. We tested whether amino acid fermentation contributed to the persistence of *C. difficile* in antibiotic-treated communities. *C. difficile* mutants unable to use proline as an electron acceptor in Stickland fermentation due to disruption of proline reductase (*prdB*−) had significantly lower levels of colonization than wild-type strains in four of six antibiotic-treated communities tested. The inability to ferment glycine or leucine as electron acceptors, however, was not sufficient to limit colonization in any communities. The data provide further support for the importance of bile salt-independent mechanisms in regulating the colonization of *C. difficile*.

**IMPORTANCE:**

*Clostridioides difficile* is one of the leading causes of hospital-acquired infections and antibiotic-associated diarrhea. Several potential mechanisms through which the microbiota can limit *C. difficile* infection have been identified and are potential targets for new therapeutics. However, it is unclear which mechanisms of *C. difficile* inhibition represent the best targets for the development of new therapeutics. These studies demonstrate that in a complex *in vitro* model of *C. difficile* infection, colonization resistance is independent of microbial bile salt metabolism. Instead, the ability of *C. difficile* to colonize is dependent upon its ability to metabolize proline, although proline-dependent colonization is context dependent and is not observed in all disrupted communities. Altogether, these studies support the need for further work to understand how bile-independent mechanisms regulate *C. difficile* colonization.

## INTRODUCTION

*Clostridioides difficile* is one of the leading causes of nosocomial infections due to its transmissibility as environmentally resistant spores and its ability to infect patients who have been treated with antibiotics (1, 2). Many classes of antibiotics can disrupt the colonic microbiota (3–5); disruption can decrease the production of inhibitory metabolites (6–9) and reduce the competition for limiting nutrients (10–13), providing favorable conditions for *C. difficile* infection.Although multiple mechanisms for colonization resistance have been identified, an understanding of the hierarchical importance of these mechanisms in *C. difficile* colonization and disease is just beginning to emerge.

Microbial bile salt metabolism has been extensively studied for its role in limiting *C. difficile* colonization. The primary bile salts cholate and chenodeoxycholate are synthesized in the liver and are conjugated to glycine or taurine to improve solubility (14). Once secreted into the intestine, microbial enzymes begin modifying these bile salts, removing conjugated amino acids and dehydroxylating primary bile salts into secondary bile salts (14). *C. difficile* spore germination is stimulated by cholate-family bile salts (cholate, taurocholate, glycocholate, and deoxycholate) and inhibited by chenodeoxycholate-family bile salts (15, 16), although there is variation in these responses between strains (17). Germination is enhanced by amino acids (15, 18) and calcium co-germinants, which act synergistically with bile salts to enhance germination (19). Secondary bile salts (deoxycholate and lithocholate) inhibit the growth of vegetative *C. difficile in vitro* (7, 15, 20), and low levels of secondary bile salts correlate with *C. difficile* infection in humans (21, 22) and mouse models (7, 23, 24).

However, recent studies have demonstrated that our understanding of the role of bile salts in *C. difficile* colonization resistance may be incomplete. *C. difficile* spore colonization and fulminant disease were observed in *Cypb8b1*^−/−^ mice unable to make cholate-family bile salts (25), and resistance to *C. difficile* colonization was observed even though these mice do not produce secondary bile salts such as deoxycholate or lithocholate. *Clostridium scindens*, a microbe inferred to inhibit *C. difficile* growth through dehydroxylation of primary bile salts to secondary bile salts (24), was also shown to inhibit *C. difficile in vitro* through the production of tryptophan-derived antibiotics (9) and other uncharacterized metabolites (26) and to potentially compete with *C. difficile* for nutrients required for growth in *Cypb8b1*^−/−^ monocolonized mice (25). In addition, both cholate and deoxycholate were shown to induce similar *C. difficile* stress responses, although 10× higher concentrations of cholate were used to observe these effects (27).

Competition for nutrients between commensal microbes and *C. difficile* has long been postulated as a mechanism for colonization resistance, with Wilson and colleagues utilizing continuous-flow culture systems to demonstrate competition between the microbiota and *C. difficile in vitro* (28, 29). *C. difficile* can metabolize mucin monosaccharides *in vitro* (30), and preferentially expresses pathways for the degradation of mucin monosaccharides in mouse models (31, 32), indicating that the metabolism of mucin monosaccharides may be a niche open to *C. difficile* during infection. Similarly, *C. difficile* metabolism of carbohydrates and sugar alcohols from the host and its diet may be another niche open to *C. difficile* during infection, as expression of genes in these metabolic pathways increases during infection in germ-free and antibiotic-treated mice (11, 31, 32). In addition to carbohydrate metabolism, *C. difficile* also efficiently utilizes amino acids through Stickland fermentation (33), which is a metabolic pathway limited primarily to proteolytic clostridial species (34), providing a unique metabolic niche within the GI tract. Increasing evidence from human (10, 35) and mouse (11, 13, 25, 31, 32, 36) studies points to Stickland fermentation with proline as an electron acceptor as a preferred nutritional niche for *C. difficile* in the GI tract. *C. difficile* can also use glycine (33) and leucine (37) as electron acceptors in Stickland fermentation, with a glycine reductase mutant recently shown to delay morbidity in a hamster model of disease (38). Multiple regulatory pathways converge to coordinately regulate the expression of proline, glycine, and leucine reductase pathways (34), and more work is needed to understand how the integration of these pathways contributes to disease in different environmental and nutritional contexts.

Previously, we described an *in vitro* minibioreactor array (MBRA) model for the characterization of *C. difficile* colonization resistance in the presence of human fecal microbial communities under nutritional conditions that simulate the distal colon (39), which has subsequently been used to characterize microbes that inhibit *C. difficile* colonization and/or toxin production (40–42). In this study, we used the MBRA model to investigate how treatment with 6 clinically used antibiotics differentially impacted susceptibility to *C. difficile* infection in microbial communities cultivated from 12 healthy individuals. As expected, we observed that antibiotic treatment reduced microbial richness and altered microbial diversity, although the extent of antibiotic-mediated disruption varied across individual communities and was not well correlated with susceptibility to *C. difficile* infection. Antibiotic treatment also reduced the microbial metabolism of cholate to deoxycholate, although there was no correlation between deoxycholate levels and susceptibility to *C. difficile* infection. To further test whether bile salts contributed to *C. difficile* colonization resistance, we cultivated fecal communities in the presence and absence of bovine bile. Similar to what was observed by Aguirre et al. (25) in *Cypb8b1*^−/−^ mice, we observed bile salts were not required for *C. difficile* colonization resistance and that the low level of *C. difficile* spore germination that occurred in the absence of bile was sufficient to allow colonization of antibiotic-treated communities by *C. difficile* spores. Using *C. difficile* mutants unable to utilize proline, glycine, or leucine as electron acceptors during Stickland fermentation due to a mutation in proline reductase (*prdB* [43]) glycine reductase (*grdA*)*,* or 2-hydroxyisocaproate dehydrogenase (*ldhA*), we demonstrated that proline reduction was required for *C. difficile* to colonize a subset of antibiotic-disrupted microbial communities, whereas glycine and leucine reduction was not required for *C. difficile* colonization in the same communities. These results can serve as a foundation for further mechanistic characterization of the hierarchical importance of different nutritional environments in limiting *C. difficile* colonization.

## RESULTS

### Loss of microbial richness is not sufficient to promote *C. difficile* colonization

**Fig 1:**
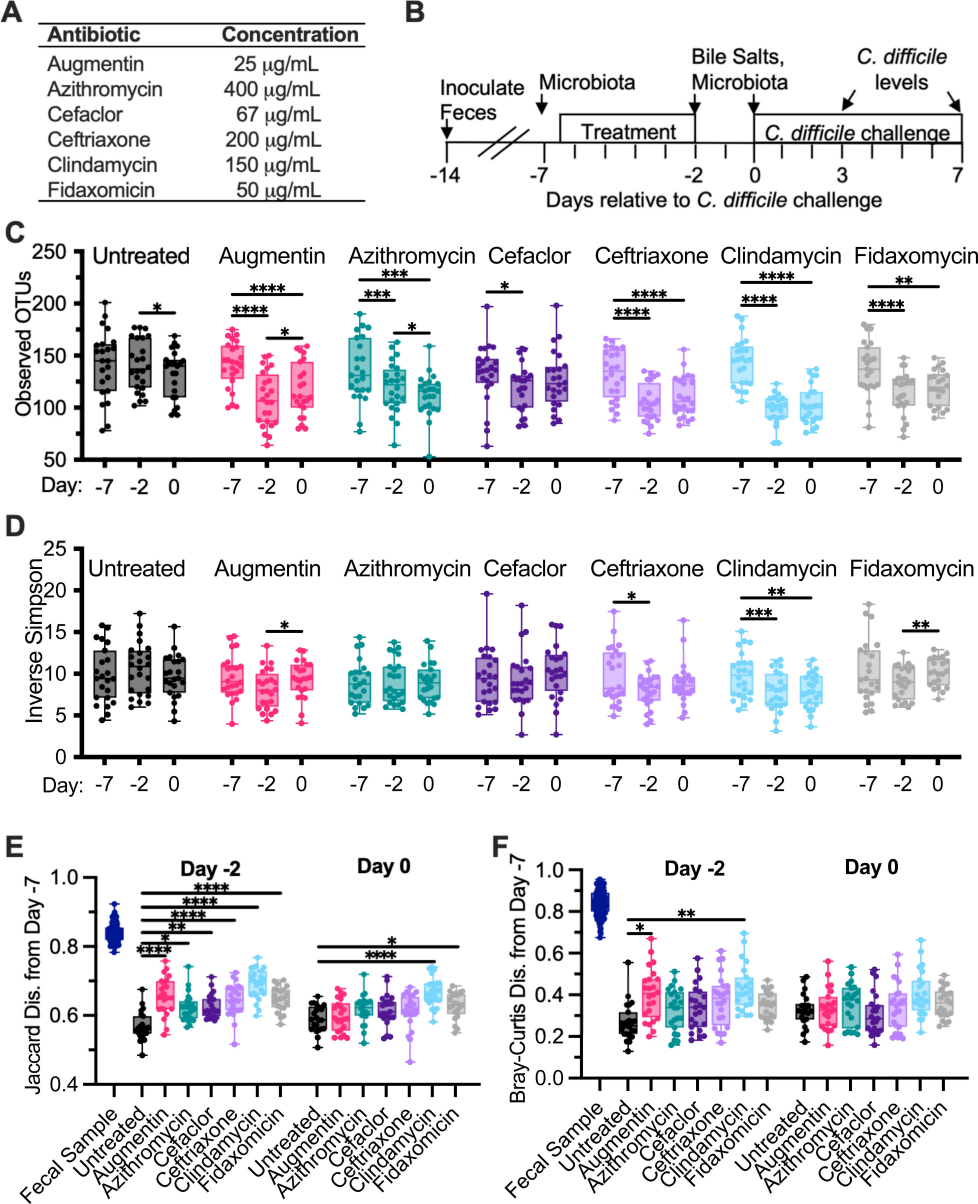
Alterations in microbiota diversity varied by type of antibiotic administered. (**A**) Antibiotics that were administered during the experiment. (**B**) Experimental timeline indicating points where samples were collected; time is indicated relative to the point of *C. difficile* challenge (day 0). In panels **C**–**F**, samples were collected from duplicate reactors inoculated with 1 of 12 healthy human fecal samples and treated as indicated. (**C**) Microbiota richness (observed operational taxonomic units [OTUs] with >99% identity) and (**D**) microbial diversity (inverse Simpson measure) were determined from 16S rRNA gene sequences. Statistical significance of changes between time points (days −7, −2, and 0) are indicated for each antibiotic. (**E**) Jaccard and (**F**) Bray-Curtis dissimilarities were determined from samples collected on day −7 and plotted for each reactor and the fecal inocula. Statistical significance of differences between each antibiotic-treated sample and untreated communities at each time point is indicated. *P < 0.05; **P < 0.01; ***P < 0.001; ****P < 0.0001. All data are plotted, with boxes representing the interquartile range (IQR) and cross lines representing the median values.

Previously, we described an *in vitro* model for culturing microbial communities from fecal samples under conditions that simulated the nutritional environment of the distal colon (44). Communities were shown to maintain a subset of microbial richness and diversity found in the starting fecal samples, similar to other *in vitro* models (44) and germ-free animal models colonized with human feces (45, 46). Furthermore, communities cultured from most healthy individuals demonstrated resistance to colonization by *C. difficile*, which could be disrupted by treatment with clindamycin (39, 41). To better understand how this model can be used to measure the effects of antibiotics on microbiota disruption and *C. difficile* colonization, we compared treatment with 6 individual antibiotics (Fig. 1A) on microbial community composition and *C. difficile* susceptibility of communities cultured from 12 different healthy individuals. These antibiotics were selected because they are all used clinically, but vary in their therapeutic spectrum of antimicrobial activity.

Fourteen independent bioreactors were inoculated with each fecal sample and allowed to stabilize in continuous culture for 7 days. Reactors were treated in duplicate with one of six antibiotics twice daily for 5 days (Fig. 1B); two reactors served as untreated controls. Two days after the end of antibiotic treatment, communities were challenged with vegetative cells of *C. difficile* strain 2015 (CD2015), a ribotype 027 clinical isolate (39). Samples were collected for analysis of microbial community composition by 16S rRNA gene sequencing from three replicates of the initial fecal sample prior to inoculation, and from cultured communities prior to administration of antibiotics (day −7), at the end of antibiotic treatment (day −2), and 2 days later (day 0), just prior to *C. difficile* challenge (Fig. 1A). Samples were collected from cultured communities for analysis of *C. difficile* levels on days 3 and 7 post-infection.

Prior to antibiotic treatment (day −7), there were no differences in richness (observed operational taxonomic units [OTUs] with ≥99% average nucleotide identity; Fig. S1A) or microbial diversity (inverse Simpson; Fig. S1B) between treatment groups, although there was a loss in richness and diversity compared to the fecal sample inocula as reported previously (44) (Fig. S1A and B). Communities were composed primarily of taxa from the Bacteroidetes, Firmicutes, Proteobacteria, and Verrucomicrobia phyla, with members of Fusobacteria, Synergistetes, and Actinobacteria phyla found in some communities (Fig. S2).

Treatment with antibiotics significantly reduced microbiota richness at the end of antibiotic treatment (day −2) relative to the baseline sample collected from the same reactor on day −7 (Fig. 1C) and led to significantly lower levels of richness compared to untreated controls on day −2 (Fig. S1A); decreases ranged from 1.1-fold (cefaclor) to 1.5-fold (clindamycin). Microbial diversity declined significantly from baseline to the end of antibiotic treatment with just two antibiotics—clindamycin and ceftriaxone (day −2; Fig. 1D). These antibiotics also showed significantly lower levels of diversity compared to untreated samples at day −2, as did the Augmentin-treated communities (Fig. S1B).

We also measured changes in shared community composition (Fig. 1D) and structure (Fig. 1E and F) by calculating Jaccard and Bray-Curtis dissimilarity measures from communities before antibiotic treatment (day −7) to communities after antibiotic treatment (day −2). (Jaccard and Bray-Curtis dissimilarity measures compare the proportion of taxa that are shared between two communities, with Jaccard providing an unweighted measure of dissimilarity between communities and Bray-Curtis providing a measure of dissimilarity that is weighted based on taxa abundance. In both cases, values closer to 0 are more similar.) As had been reported previously (44), cultivation led to significant shifts in microbial composition and structure from initial fecal samples (Fig. 1E and F) to day −7. Following this initial reorganization in community composition and structure, continued cultivation led to lower levels of change in community composition and structure (Fig. 1E and F, see untreated samples on day −2 and day 0). Treatment with all antibiotics led to larger changes in community composition from day −7 to day −2 compared to untreated communities (Fig. 1E), whereas only Augmentin and clindamycin also led to significantly larger changes in community structure compared to untreated communities (Fig. 1F).

Two days following the end of antibiotic treatment (day 0), reduced richness compared to baseline samples persisted for all antibiotic-treated communities with the exception of those treated with cefaclor (Fig. 1C), although Augmentin-treated communities exhibited small, but statistically significant increases in microbial richness (Fig. 1C). Augmentin-treated communities also exhibited small, but statistically significant increases in microbial diversity (Fig. 1D) and decreases in Jaccard (Fig. S1E) and Bray-Curtis (Fig. S1F) dissimilarity, indicating a potential return toward baseline following cessation of this antibiotic. Clindamycin-treated communities continued to exhibit decreased microbial diversity (Fig. 1D) and less similar community composition to baseline (Fig. 1E).

**Fig 2:**
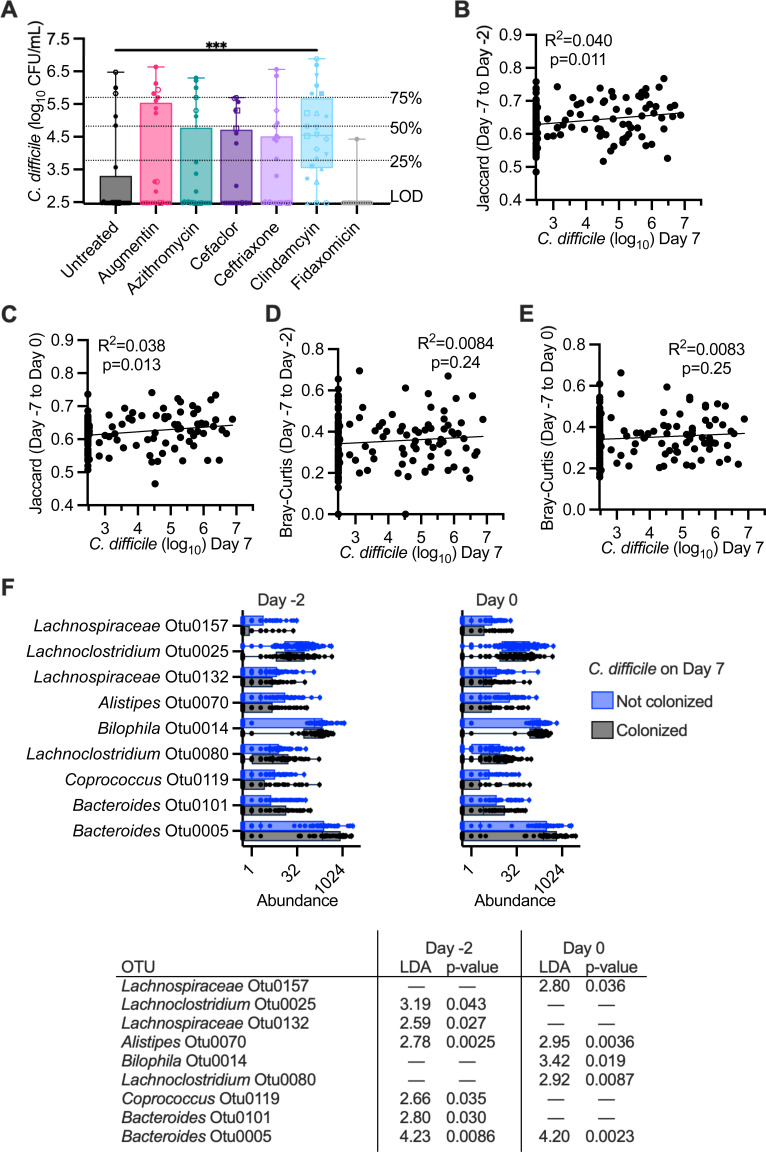
Clindamycin treatment increases susceptibility to *C. difficile* colonization. (**A**) Levels of *C. difficile* were measured in all reactors on day 7 of infection. Data are pooled by treatment, with each reactor and replicate indicated by a distinct symbol consistent across treatment groups (effects on individual fecal samples can be better visualized in Fig. S3). The statistical significance of differences between each antibiotic-treated community and untreated communities are indicated; ***P < 0.001. (**B**–**E**) Simple linear regression between levels of *C. difficile* on day 7 and Jaccard (**B and C**) and Bray-Curtis (**D and E**) dissimilarity on day −2 (**B and D**) or day 0 (**C and E**). (**F**) OTUs that differed significantly in abundance on day −2 and/or day 0 in communities that were colonized or not colonized with *C. difficile* on day 7 were identified with LEfSe analysis of rarefied OTU abundance data. The abundance of these OTUs across all samples is plotted for colonized and not colonized communities, with linear discriminant analysis (LDA) and *P*-values determined by LEfSe reported below abundance plots.

*C. difficile* susceptibility, measured as levels of *C. difficile* detected on day 7 following challenge with *C. difficile* cells, varied across different antibiotic treatments (Fig. 1E) and fecal donors (Fig. S3). While *C. difficile* levels on days 3 and 7 were collected, we used levels of *C. difficile* on day 7 rather than day 3 as a marker of colonization to provide sufficient time for loss of non-replicating cells and spores under continuous flow culture conditions. Of the 12 fecal samples tested, untreated communities from 6 fecal samples were resistant to colonization, defined as *C. difficile* colony forming units (CFU)/mL undetectable in both replicates, and untreated communities from one fecal sample were colonized at high levels, with both replicates colonized with *C. difficile* levels in the highest quartile. The remaining five untreated communities exhibited variable susceptibility to colonization (Fig. S3A), with two fecal communities exhibiting low (1st quartile) or undetectable levels of colonization in both replicates. Clindamycin was the only antibiotic that significantly increased median levels of *C. difficile* colonization above levels observed in untreated communities (Fig. 2A); 10 of 12 fecal sample communities were colonized following treatment with clindamycin (Fig. S3A). Susceptibility to colonization varied across communities from different fecal samples that were treated with other antibiotics (Fig. S3A).

We tested whether there were correlations between levels of richness or microbial diversity on day −2 or day 0 and found that there were no significant correlations with *C. difficile* colonization levels (Fig. S4). We also assessed whether changes in community composition (Jaccard dissimilarity) or structure (Bray-Curtis dissimilarity) from day −7 to day −2 or day 0 correlated with *C. difficile* colonization levels. We observed weak correlations with changes in microbial composition from baseline (day −7) to day −2 (Fig. 2B) and day 0 (Fig. 2C). There were no significant correlations with changes in community structure from baseline to day −2 or day 0 (Fig. 2E). Similar results were observed for *C. difficile* levels on day 3 (Fig. S5). We used LEfSe (47) to identify specific OTUs whose abundance on day −2 or day 0 correlated with *C. difficile* colonization. We found a small number of OTUs whose abundance on day −2 or day 0 significantly correlated with *C. difficile* levels (Fig. 2F), with four OTUs more abundant in non-colonized communities on day −2 and/or day 0 and five OTUs more abundant in colonized communities on day −2 and/or day 0.

### Antibiotic treatment alters bile salt metabolism

**Fig 3:**
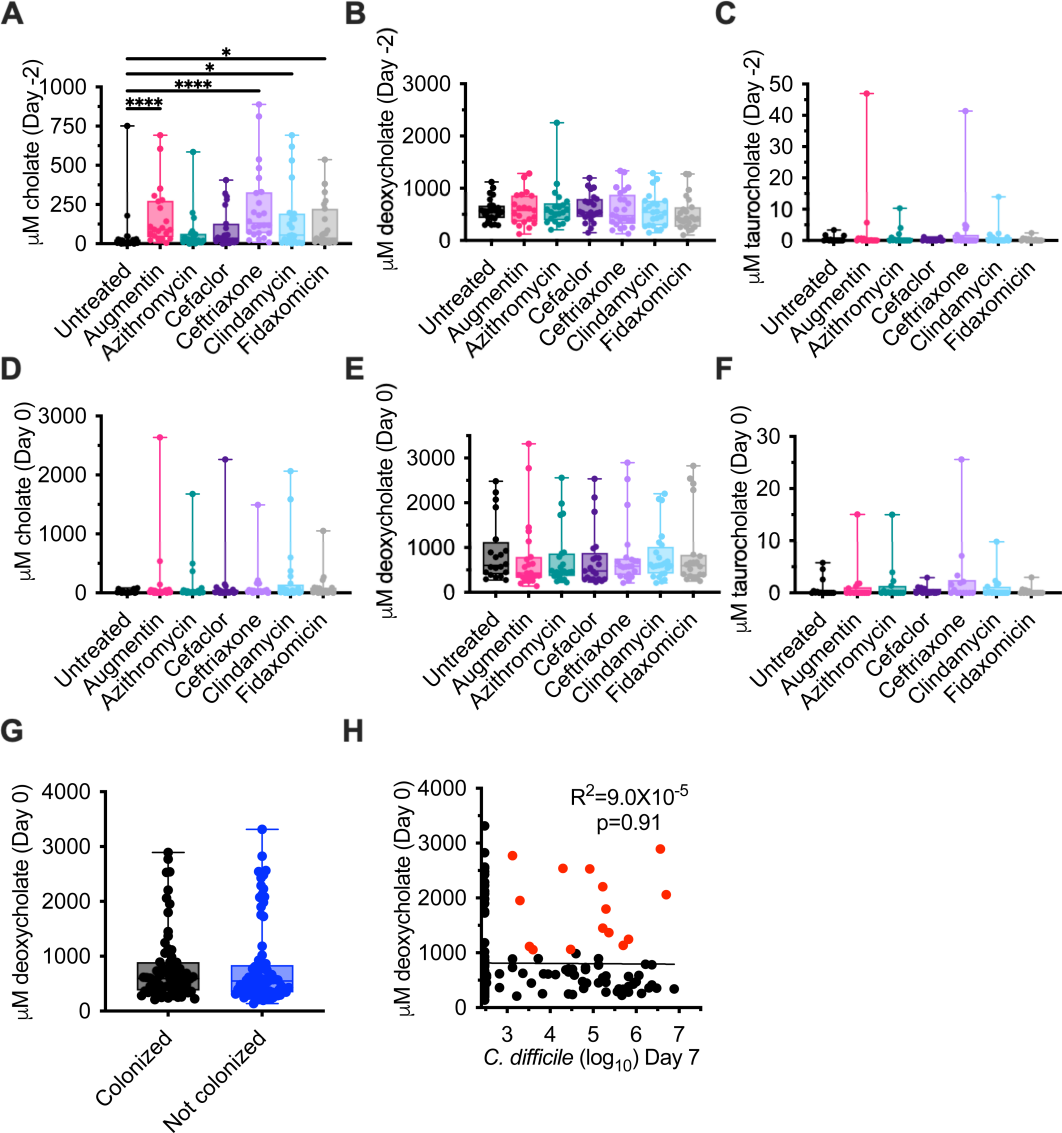
Cholate, deoxycholate, and taurocholate levels in untreated and antibiotic-treated bioreactors. Levels of (**A, D**) cholate, (**B, E**) deoxycholate, and (**C, F**) taurocholate were measured from the communities described in Fig. 1 at the end of antibiotic treatment (day −2; **A–C**) and just prior to *C. difficile* challenge (day 0; **D–F**). Significance of differences between antibiotic-treated and untreated samples at each time point is reported. *P < 0.05; **P < 0.01; ***P < 0.001; ****P < 0.0001. (G) Levels of deoxycholate are plotted for the communities identified as colonized and not colonized in Fig. 2. (H) Simple linear correlation between levels of deoxycholate measured on day 0 and *C. difficile* levels on day 7. Colonized communities with deoxycholate levels greater than 1 mM are indicated in red.

Bioreactor medium (BRM3) contains bovine bile as a complex source of bile salts. Analysis of the bioreactor medium indicated that cholate family bile salts predominated, with approximately 36% taurocholate (198 µM), 34% glycocholate (190 µM), 10% cholate (56 µM), and 2% deoxycholate (13 µM) of total bile salts measured (Fig. S6A and B). To understand how microbiota cultivation and antibiotic treatment altered bile salt pools in bioreactor cultures, we focused on quantification of the amino acid conjugated primary bile salt, taurocholate, the primary bile salt, cholate, and the secondary bile salt, deoxycholate across the communities described in Fig. 1. We observed that microbiota cultivation led to a >6,500-fold decrease in median levels of taurocholate and a 45-fold increase in median levels of deoxycholate in spent culture supernatant in untreated communities on day −2 compared to fresh medium (Fig. S6C). Treatment with four antibiotics—Augmentin, ceftriaxone, clindamycin, and fidaxomicin—led to significantly higher levels of cholate compared to untreated communities at the end of antibiotic treatment (day −2; Fig. 3A). Deoxycholate and taurocholate levels were not significantly different between untreated communities and communities treated with antibiotics on day −2 (Fig. 3B and C). There were no significant differences in bile salt levels between untreated and antibiotic-treated communities on day 0 (Fig. 3D through F). There was no correlation between levels of C. difficile on day 7 and cholate levels on day −2 (Fig. 3G) or day 0 (*R*^2^ = 8.5 × 10^−8^, *P* = 0.997).

Previous studies have found that deoxycholate inhibits the growth of vegetative *C. difficile* cells (7, 15, 20), with inhibitory concentrations ranging from 0.01% to 0.1% (250–2,500 µM). The median concentration of deoxycholate measured in untreated communities on day 0, the day of the *C. difficile* challenge, was 602 µM (Fig. 3B); median concentrations in antibiotic-treated communities ranged from 408 µM (Augmentin) to 608 µM (clindamycin), but were not significantly different than those observed in untreated communities (Fig. 3E). Because both *C. difficile* susceptibility and deoxycholate levels varied by fecal donor and antibiotic treatment (Fig. S7), we tested whether deoxycholate levels on day 0 correlated with *C. difficile* colonization. We observed that there were no significant differences in levels of deoxycholate between colonized and uncolonized communities (Fig. 3H), nor were there significant correlations between deoxycholate levels and *C. difficile* levels on day 7 (Fig. 3I). In addition, we observed that 21.4% of colonized communities (15/70) had deoxycholate levels >1,000 μM on day 0 (Fig. 3I, symbols colored red).

### Bile was not required for *C. difficile* colonization resistance *in vitro*

**Fig 4:**
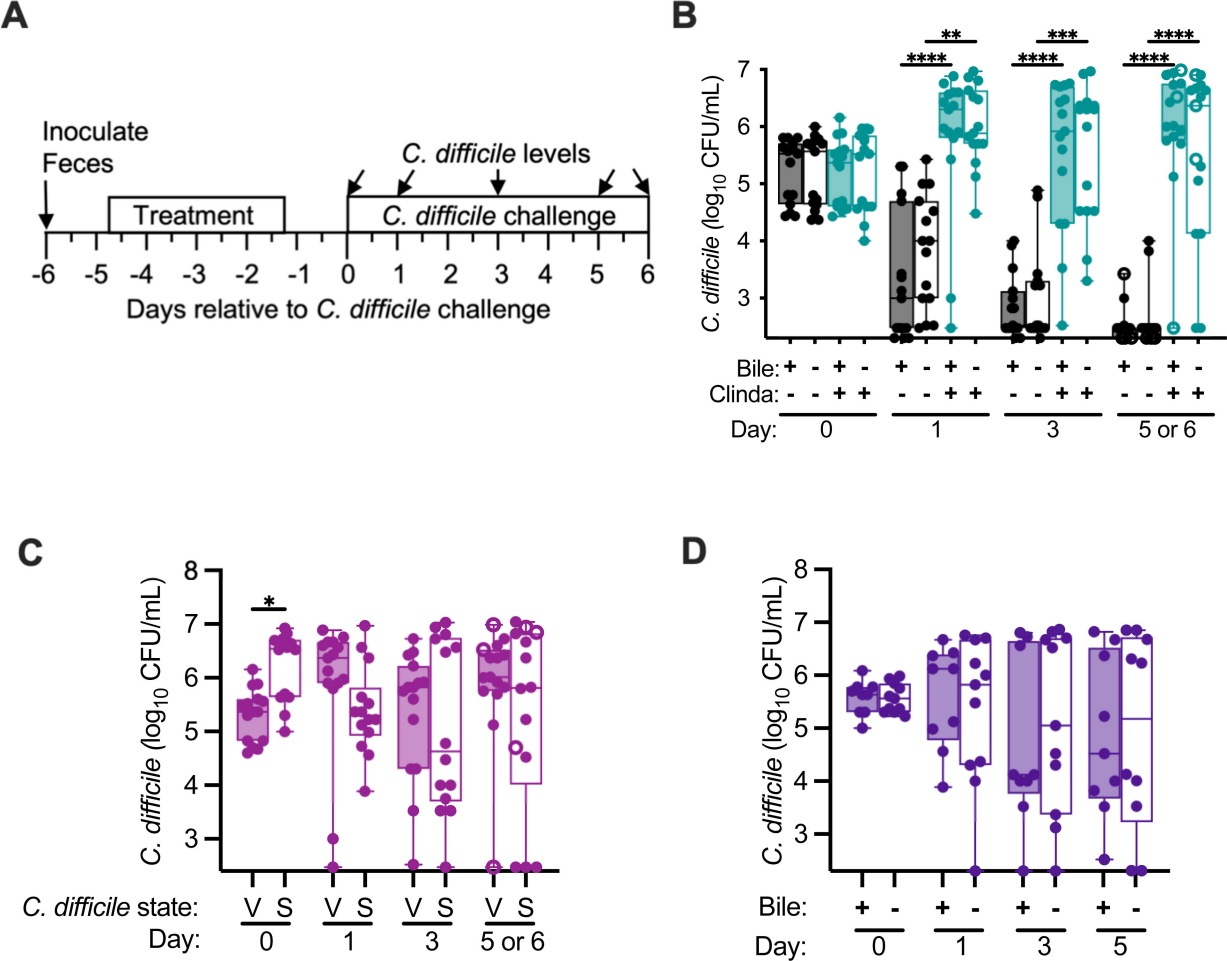
Bile acids were not required for colonization resistance and did not enhance colonization in communities of human fecal microbes cultured in bioreactors. (**A**) Experimental timeline indicating points where samples were collected; time is indicated relative to the point of *C. difficile* challenge. The final collection timepoint was on day 5 for all samples with the exception of FS22, which was collected on day 6 and is represented by open symbols. (**B**) *C. difficile* levels in untreated and clindamycin-treated communities cultured in the presence and absence of bile. The significance of differences between untreated and clindamycin-treated samples is shown. *P < 0.05; **P < 0.01; ***P < 0.001; ****P < 0.0001. (**C**) *C. difficile* levels in clindamycin-treated communities challenged with *C. difficile* vegetative (V) cells or spores (S). The significance of differences between vegetative and spores is shown at each time point. (**D**) *C. difficile* levels in clindamycin-treated communities cultured in the presence or absence of bile challenged with *C. difficile* spores. No significant differences were observed between communities cultured in the presence or absence of bile.

While the previous results were consistent with the hypothesis that secondary bile salts do not mediate colonization resistance in communities cultured in bioreactors, we could not rule out the effects of other bile salts that were not measured. To test whether bile salts were required, we monitored the colonization resistance of microbial communities established from five fecal samples cultured in parallel in media with and without added bile. Specifically, replicate bioreactors were established for each fecal sample in media either containing or lacking bovine bile. Triplicate communities were treated with clindamycin as previously described (48), while the remaining three replicates for each type of media were left untreated (Fig. 4A). Communities were challenged with vegetative cells of CD2015 and *C. difficile* levels were monitored over time. We found *C. difficile* colonization was similarly suppressed in communities cultured in the presence or absence of bile and that treatment with clindamycin led to similar levels of *C. difficile* colonization (Fig. 4B).

### Bile was not required for *C. difficile* spores to colonize *in vitro*

Cholate family bile salts are known to greatly enhance *C. difficile* spore germination during *in vitro* culture (15, 49, 50), although they are not required for spore germination in a mouse model of infection (25). Previously, we reported that we were unable to colonize a clindamycin-treated microbial communities cultured in an MBRA model with *C. difficile* spores (39). Because these studies were performed using a microbial community formed from pooling 12 fecal donors, we tested the ability of spores to colonize clindamycin-treated communities established from fecal samples collected from five individuals. We found that the majority of clindamycin-treated communities could be colonized following challenge with *C. difficile* spores (Fig. 4C), although communities from one fecal sample were colonized at a lower frequency by spores than by vegetative cells (Fig. S8). We then tested whether bile salts were required for *C. difficile* spores to colonize clindamycin-treated fecal microbial communities cultured in MBRAs. We observed that colonization by spores was not significantly different between communities cultured in media with and without bile (Fig. 4D).

**Fig 5:**
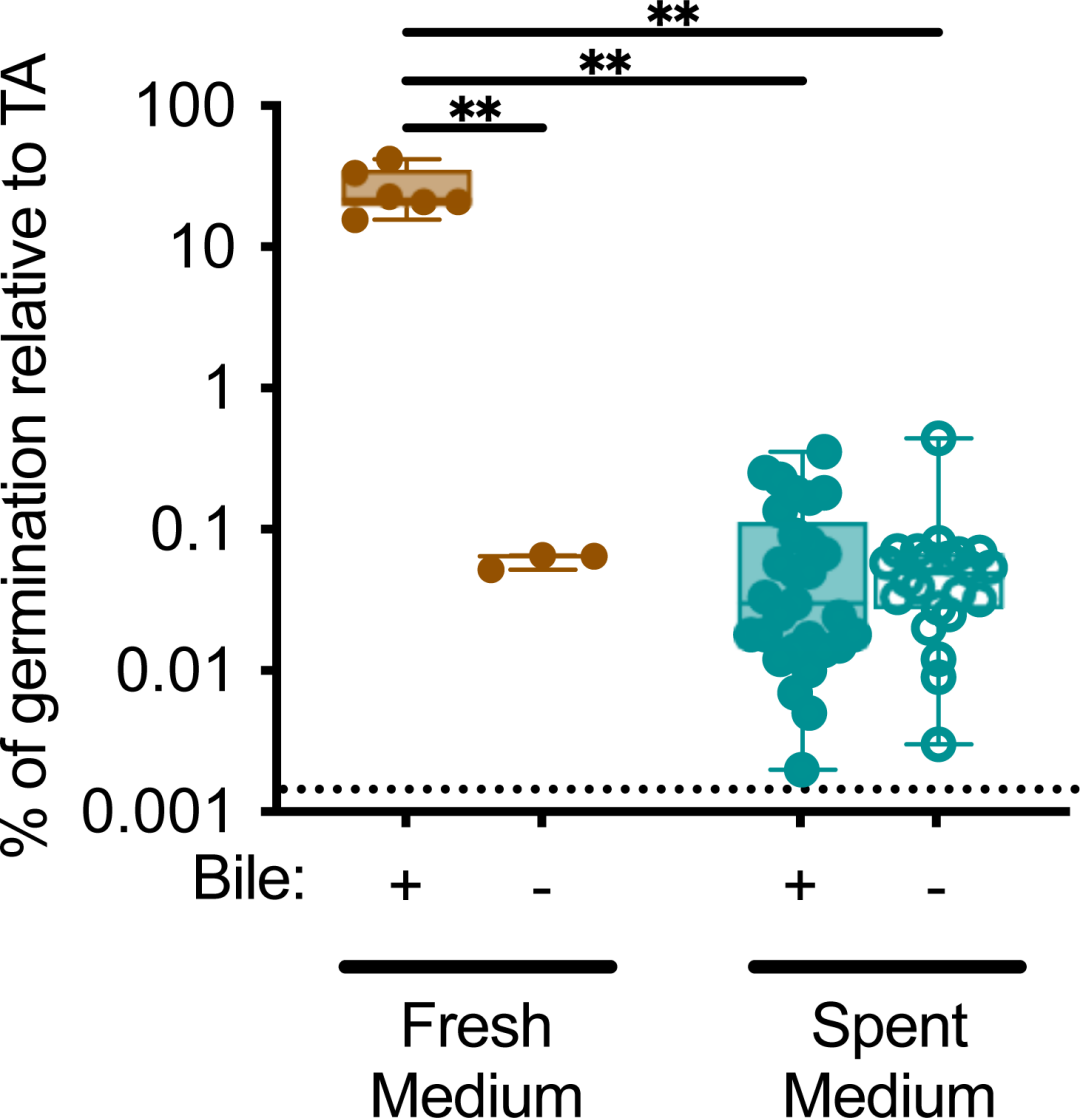
Low levels of spore germination in spent culture medium from communities cultured in the presence of bile. Germination of CD2015 spores was determined in filter-sterilized spent culture medium from clindamycin-treated fecal communities cultured in the presence or absence of bile. Spores were incubated in medium for 1 hour followed by enumeration on agar medium with or without added taurocholate. The percent germination on taurocholate indicates the percent of colonies recovered after overnight incubation in the absence of taurocholate compared to incubation in the presence of taurocholate for each sample. Fresh bioreactor media made with or without bovine bile were included as controls. Significant difference observed in comparisons between all samples is reported. ns, *P* > 0.05; **P < 0.01; ***P < 0.001.

Because cholate family bile salts were previously reported to enhance germination, we compared the ability of media with and without bile to enhance the colony formation of spores in pure culture. Consistent with previous observations (15, 49), incubation of CD2015 spores in bioreactor medium containing bile for 1 hour enhanced colony formation by germinated *C. difficile* spores by >400-fold compared to incubation in the presence of bioreactor medium without bile (Fig. 5). We next tested how spent culture medium collected from clindamycin-treated communities cultured in the presence or absence of bile would impact spore germination. Low levels of spore germination were observed in spent culture medium from fecal communities cultured in the presence and absence of bile (Fig. 5). Altogether, the data indicate that the levels of bile salts present in spent culture medium from communities cultured in the presence of bile do not have significant impacts on germination. Furthermore, while the levels of spore germination and colony formation in the spent culture medium are low, these low levels of germination would result in ~10^3^ vegetative cells, a level previously shown to infect antibiotic-disrupted communities (39).

### Proline metabolism was required for *C. difficile* to colonize a subset of fecal samples *in vitro*

Proteolytic metabolism through Stickland fermentation has previously been reported as an important nutritional niche for *C. difficile* in humans (10, 35) and animal models of infection (11, 13, 25, 31, 32, 36, 38). As the bioreactor medium contains low levels of fermentable carbohydrates and does not contain mucin-associated monosaccharides, we hypothesized that Stickland fermentation with proline as an electron acceptor could be important for the colonization of antibiotic-disrupted fecal communities. To test this hypothesis, we compared the colonization of mutants defective in the ability to metabolize proline (*prdB*::CT [43]) to a wild-type (wt) strain; both wild type and *prdB*::CT were in the CD196 background, which is a non-epidemic ribotype 027 isolate (51). Fecal samples from six healthy individuals were cultured in replicate bioreactors, treated with clindamycin, and challenged with wt or *prdB*::CT strains using the approach described in Fig. 4A.

**Fig 6:**
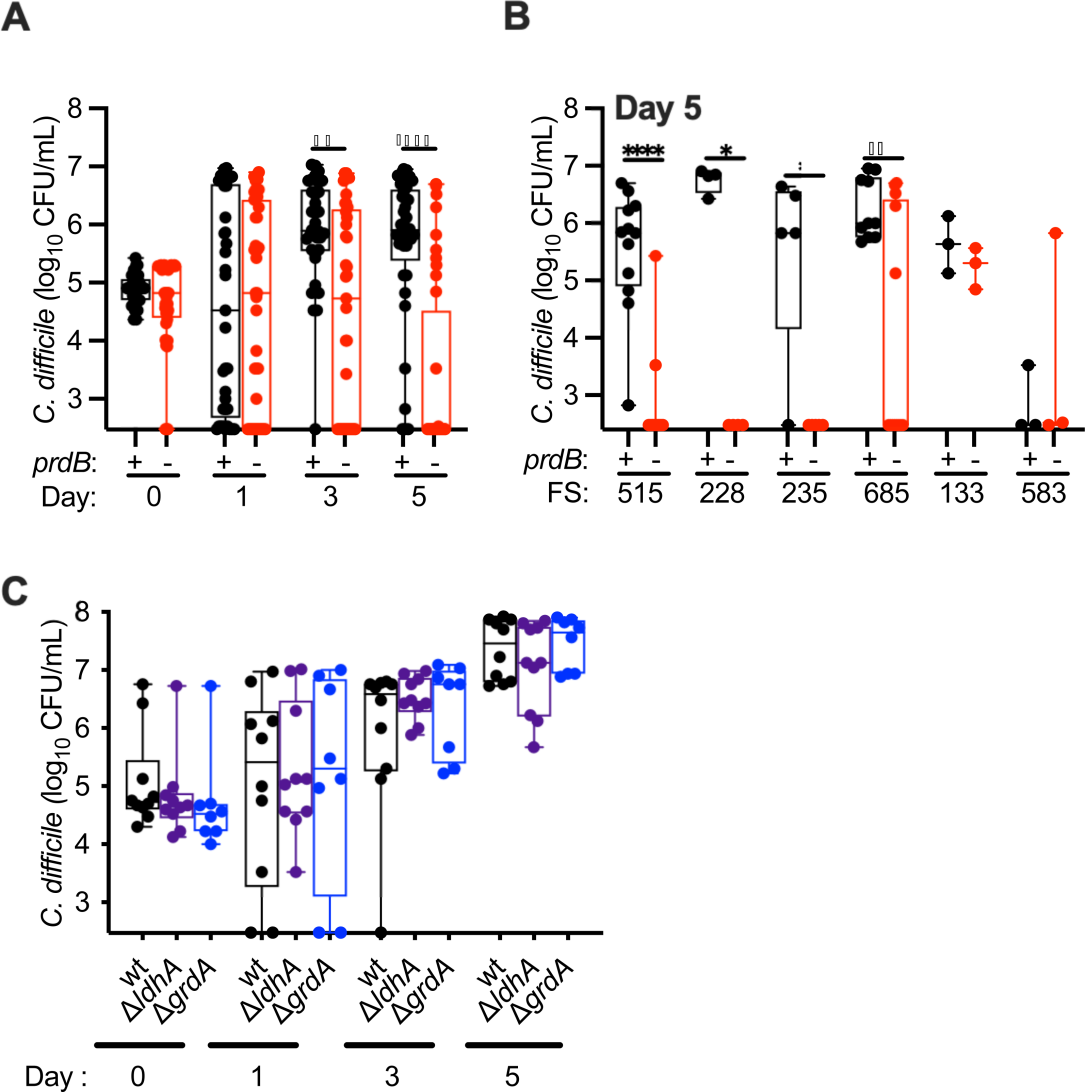
*C. difficile prdB*::CT mutants fail to persist in most clindamycin-treated communities. (**A**) Levels of wt (*prdB*^+^) and *prdB*::CT (*prdB*^−^) CD196 strains were measured over time following introduction into fecal communities treated with clindamycin as outlined in Fig. 4A. Data reported are pooled from all six fecal samples tested. Significant differences between wild-type and mutant strains at each time point are reported. ***P < 0.001; ***P < 0.0001. (**B**) Levels of wt and *prdB*::CT CD196 strains were measured on day 5 of colonization from each of the six fecal samples tested (FS515, FS228, FS235, FS685, FS133, and FS583). Significant differences between wild-type and mutant strains for each fecal sample are reported. *P < 0.05; **P < 0.01; ****P < 0.001. (**C**) Levels of wt, Δ*ldhA*, and Δ*grdA* R20291 strains were measured over time following introduction into fecal communities treated with clindamycin as outlined in Fig. 4A. No significant differences between wild-type and mutant strains were observed at any time point.

Overall, we observed that fecal communities challenged with *prdB* mutants showed decreasing levels of colonization over time, with significantly lower levels observed on days 3 and 5 of colonization compared to wild type (Fig. 6A). However, the ability of wt and *prdB*::CT strains to colonize was dependent upon the fecal community tested (Fig. 6B). Wild-type strains colonized five of the six clindamycin-disrupted communities (FS515, FS228, FS235, FS685, and FS133), whereas *prdB*::CT strains failed to colonize two of these susceptible communities (FS228 and FS235). In the other three susceptible communities, there was either a lack of *prdB*::CT colonization in the majority of replicates tested (FS515: 10/12 replicates; FS685; 8/13 replicates), or there was no difference between levels of wt and *prdB*::CT colonization levels (FS133). Based on these results, we conclude that Stickland fermentation with proline as an electron acceptor can play an important role in the ability of *C. difficile* to persist in complex communities *in vitro*, but this is dependent upon the composition of the fecal community and its ability to limit *C. difficile* colonization through other mechanisms.

As both glycine and leucine can also serve as electron acceptors for Stickland fermentation, we hypothesized that fermentation of glycine or leucine may also contribute to persistence in clindamycin-treated communities. We tested the ability of mutants defective in Stickland fermentation of glycine and leucine due to mutations in glycine reductase (Δ*grdA*) and 2-hydroxyisocaproate dehydrogenase (Δ*ldhA*), respectively, to persist in clindamycin-treated communities. Persistence of these mutants, generated in the hypervirulent ribotype 027 R20291 background, was compared to R20291 wild-type strains in four of five fecal samples tested in Fig. 6A (FS515, FS228, FS235, and FS133). In these studies, we saw no significant differences in colonization levels between wild-type and mutant strains over time, indicating that defects in glycine or leucine fermentation may not be sufficient to limit *C. difficile* colonization in this model.

## DISCUSSION

The factors that govern whether or not an individual exposed to *C. difficile* will go on to develop symptomatic disease are complex. While it is clear that disruption of the GI microbiota is a key risk factor for infection and disease, the extent to which different microbes interact with each other and the host to limit *C. difficile* infection and disease progression is not completely understood. Developing tools that allow microbe and metabolite interactions to be investigated in the absence of a host can help to provide insights into whether mechanisms that inhibit or promote *C. difficile* colonization are more likely to be causative or correlative. This level of understanding is necessary as new therapeutic approaches that more narrowly target *C. difficile,* such as defined microbial consortia, are developed.

To gain greater insights into factors that govern *C. difficile* colonization resistance in a fecal MBRA model, we investigated how six different clinically used antibiotics impacted the ability of the microbiota to resist *C. difficile* colonization. As expected, we observed that antibiotic treatment led to the loss of microbial richness, although the extent of microbiota disruption varied both by the class of antibiotic tested and by the composition of the microbial community. Consistent with previous meta-analyses of human antibiotic exposure and risk for *C. difficile* infection in non-hospitalized patients (52, 53), clindamycin was most strongly associated with susceptibility to colonization in antibiotic-treated communities, with 10 of 12 communities tested showing susceptibility following treatment. However, the magnitude of microbiota disruption across antibiotic treatments did not correlate with susceptibility to *C. difficile* colonization, with some communities with significant losses in richness resisting *C. difficile* colonization and other communities with modest richness losses exhibiting susceptibility to colonization. The data provide further support for the hypothesis that *C. difficile* colonization resistance is likely due to the presence of specific microbes and their functions that limit *C. difficile* colonization rather than overall microbiota diversity.

Due to the compositional diversity between fecal communities tested, there were relatively few conserved taxa whose presence or absence correlated with susceptibility to *C. difficile* colonization, which is consistent with the model that differences in microbiota function rather than composition are important for understanding *C. difficile* susceptibility. Similar to previous studies (41, 54), however, levels of multiple *Lachnospiraceae* OTUs were higher in resistant communities.

To better understand which microbiota functions may be important for colonization resistance in this model, we investigated two mechanisms previously proposed to regulate *C. difficile* colonization: microbial metabolism of bile acids and Stickland fermentation with proline, glycine, or leucine as an electron acceptor. We observed that microbial communities cultured in bioreactors were able to convert the taurocholate in bovine bile into deoxycholate. Treatment with antibiotics had no significant impacts on levels of taurocholate. Microbes encoding bile salt hydrolases, which remove taurine and glycine from primary bile salts (14), are broadly distributed amongst members of the GI microbiota (55), which may have contributed to the ability of this function to persist during antibiotic treatment.

By contrast, enzymes necessary to perform 7α-dehydroxylation of cholate to deoxycholate are restricted to a smaller subset of the GI microbiota, primarily members of the Clostridiales family and the *Clostridium* genus (56). Treatment with Augmentin, ceftriaxone, clindamycin, and fidaxomicin significantly increased levels of cholate at the end of antibiotic treatment on day −2, suggesting that microbes capable of 7α-dehydroxylation of cholate to deoxycholate may be lost in some communities, although levels of deoxycholate were not significantly lower at these time points. This lack of significant differences in deoxycholate levels across all fecal sample communities may partly be due to differences in how different fecal communities responded to antibiotic treatment (Fig. S7), as some communities (e.g., FS3 and FS9) exhibited much larger decreases in deoxycholate than others (e.g., FS6 and FS12). There was also no correlation between levels of deoxycholate and susceptibility to *C. difficile* colonization, indicating that secondary bile salt-mediated inhibition was unlikely to be a mechanism preventing colonization in this model. This was further supported by experiments investigating colonization resistance in the presence and absence of bile, which demonstrated bile was not required for *C. difficile* colonization resistance.

The lack of inhibition by secondary bile salts was surprising, as levels of deoxycholate measured in spent culture medium (431–608 µM) were within the range shown to inhibit *C. difficile* growth in pure culture *in vitro* (250–2,500 µM [7, 15, 20]). Nevertheless, >20% of communities colonized with *C. difficile* had deoxycholate levels >1,000 μM on day 0 of colonization, suggesting inhibitory effects observed in pure culture may not be the same in complex culture in the presence of other microbes. Further studies are needed to understand whether the presence of other microbes could mitigate some of the toxic effects of deoxycholate on *C. difficile* growth.

We also observed that spores were capable of infecting communities treated with clindamycin and this was independent of the presence of bile. Consistent with what has been previously reported, low levels of germination were observed in the absence of bile and in spent culture medium cultured in the presence or absence of bile. However, these low levels of germination would result in ~10^3^ vegetative cells, a level previously shown to infect antibiotic-disrupted communities cultured in bioreactors (39). The data provide further support to prior studies that have proposed that control of *C. difficile* germination and growth (12, 25) through restoration of microbial bile salt metabolism may not be sufficient to limit *C. difficile* colonization.

While levels of bile salts did not correlate with susceptibility to infection, the ability of *C. difficile* to metabolize proline as an electron acceptor for Stickland fermentation was required for persistence in the majority of disrupted communities tested. These results are consistent with evidence from human (10, 35) and mouse (11, 13, 25, 31, 32, 36) studies that point to Stickland fermentation with proline as an electron acceptor as a preferred nutritional niche for *C. difficile* in the GI tract. Stickland fermentation is a metabolic pathway limited primarily to proteolytic clostridial species (34). This includes microbes such as *C. scindens*, which can compete with *C. difficile* for proline (25), dehydroxylate primary bile salts to secondary bile salts (24), and can produce a bacteriocin that limits *C. difficile* growth (9). Identifying microbes that effectively compete with *C. difficile* for proline and other amino acids following antibiotic treatment may be one approach to limit *C. difficile* colonization.

It is unclear which metabolites were supporting *C. difficile* growth in antibiotic-treated communities where *prdB* mutants persisted at higher levels over time. Stickland fermentation with glycine or leucine as an electron acceptor is one potential niche that could have been utilized by *prdB* mutants*,* although we observed the mutants in either pathway alone (Δ*grdA* and Δ*ldhA*) were not sufficient to limit *C. difficile* colonization. Recent studies in a hamster model of infection have shown that hamsters infected with Δ*grdAB* mutants in strain CD630 show delayed morbidity compared to wild-type CD630, although there were no significant differences between wt and Δ*grdAB* mutants in levels of *C. difficile* or toxin recovered from the cecum at necropsy (38), indicating the role of glycine fermentation *in vivo* may be relatively modest. Alternatively, *C. difficile* may have utilized carbohydrate fermentation pathways. Further studies are needed to more fully understand how *C. difficile* colonizes disrupted communities.

Altogether, these studies highlight the importance of improving our understanding of bile-independent mechanisms regulating *C. difficile* colonization. While it is clear that Stickland fermentation of amino acids with proline as an electron acceptor is important for *C. difficile* persistence in many disrupted communities, other nutritional niches should also be explored, including whether Stickland fermentation with both glycine and leucine may contribute to reduced persistence. Studies similar to those described here, examining variations in fecal microbial communities and environmental conditions, will further clarify the hierarchical importance of different nutritional environments in limiting *C. difficile* colonization.

## MATERIALS AND METHODS

### Strains used in this study

*C. difficile* strain 2015 (39), a fully sequenced ribotype 027 isolate resistant to rifampicin and erythromycin (accession: CP073752.1), was used for routine studies to assess *C. difficile* colonization. To test the role of reductive fermentation of proline in colonization, a previously described ClosTron mutant in proline reductase (*prdB*::ClosTron [43]) along with its congenic wild-type strain was tested. Although originally reported as mutants in the R20291 background, these strains are in the CD196 background (E. Skaar, personal communication). To test the role of reductive fermentation of glycine and leucine, deletions of the coding sequences of glycine reductase (*grdA*) and 2-hydroxyisocaproate dehydrogenase (*ldhA*) were generated by riboswitch-mediated allelic exchange in strain R20291 (57). Successful deletion of genes and absence of second site mutations was verified by Illumina sequencing. Specifically, short read, whole-genome Illumina sequencing was performed by SeqCoast Genomics (Portsmouth, NH, USA). Sequencing reads for mutant strains were both compared to the parental *C. difficile* R20291 strain by Geneious Prime’s Geneious alignment algorithm. Sequence variants were then analyzed and both strains were found to be free of mutations outside of the expected gene deletions. Sequences were deposited to NCBI SRA with accession numbers SRR31632037 and SRR31632038. These mutants were tested along with their congenic wild-type R20291 strain.

### Fecal sample collection and preparation

Fecal samples were collected from 18 healthy individuals who had not been treated with oral antibiotics within the previous 6 months. Samples were collected from children aged 4–17 (*n* = 3), adults aged 18–65 (*n* = 12), and older adults aged >65 (*n* = 2). Similar numbers of male and female participants agreed to provide samples. However, only donor age categorization (child, adult, older adult) remained linked to specific fecal samples following de-identification. Studies were designed to collect samples across the age span rather than specifically powered to evaluate differences in community composition by age. All adult participants provided consent to participate in the study. Children provided assent to participate along with parental consent to participate. Protocols for the collection and use of fecal samples were reviewed and approved by Institutional Review Boards at Baylor College of Medicine (protocol number H-38014) and the University of Nebraska-Lincoln (protocol numbers 18585 and 20186).

Samples were self-collected by participants in commode specimen collection containers, sealed in a plastic bag containing a gas pack (BBL GasPak Anaerobe sachet), packed in ice packs, placed in a sealed container, and returned to the laboratory within 24 hours as previously described (39). Fecal samples were manually homogenized and subdivided under anaerobic conditions (anaerobic chamber with 5% H_2_, 5% CO_2_, 90% N_2_ atmosphere) and frozen at −80°C until later resuspended at 25% (wt/vol) in reduced phosphate-buffered saline (PBS). Fecal suspensions were vortex-mixed for 5 min at >2,500 rpm, centrifuged at 200 × *g* for 5 min to settle large particulates, and the supernatants were either used immediately or amended with 7.5% dimethylsulfoxide and preserved at −80°C until use. Previous work had shown that freezing samples at −80°C did not significantly change the composition of communities cultured in bioreactors (44).

### Bioreactor experiments

MBRAs were assembled and operated under anoxic conditions (5% H_2_, 5% CO_2_, and 90% N_2_) at 37°C as described previously (39). MBRAs are strips of six independent continuous flow bioreactors that operate at a 15 mL volume and are continuously stirred with small magnetic stir bars positioned over a stir plate. Each independent reactor in the array contains three ports, one for delivery of fresh medium at a slow continuous rate, one for removal of waste, and a sample port for sampling of medium and bacteria in suspension. Bioreactor medium version 3 (BRM3 [58]; Table S1), which simulates nutritional conditions of the distal colon was used for all studies, with the exception of studies testing the effect of bovine bile, when this component was excluded from BRM3. Fecal suspensions from individual donors were inoculated into sterile, anoxic BRM3 medium at 1% (wt/vol) final concentration and allowed to grow for 16–24 hours in batch, prior to initiation of continuous flow of medium at 1.875 mL/hour (8 hours of retention time for 15 mL bioreactors). Communities were allowed to equilibrate under continuous flow for 1 or 6 days as indicated in the figures. Antibiotics were either administered twice daily to individual bioreactors or added directly to the source medium as indicated in the figures. Research grade antibiotics were obtained from the following sources: Augmentin (5:1 amoxicillin sodium salt to potassium clavulanate; Research Products International); azithromycin dihydrate (Thermo Scientific Chemicals); cefaclor (Thermo Scientific Chemicals); ceftriaxone sodium salt hemiheptahydrate (Thermo Scientific Chemicals); clindamycin phosphate (TCI America); and fidaxomicin (Apexbio Technology, LLC). Antibiotic concentrations used for twice-daily dosing were estimated based on previously published measurements in human feces and/or bile (59–65), although fidaxomicin dosing was limited by its maximal solubility. Clindamycin concentrations used in media were previously described (42). One or two days following cessation of antibiotics as indicated in figure legends, *C. difficile* spores or vegetative cells were administered to reactors at ~1 × 10^5^ CFU/mL (vegetative cells) or 1 × 10^6^ CFU/mL (spores), and levels were enumerated over time. Samples were collected from bioreactors at the time points indicated using a sterile needle and syringe to collect bacteria in suspension. When appropriate, an aliquot of this sample was used to enumerate *C. difficile* by serial dilution and plating on taurocholate cefoxitin cycloserine fructose agar (TCCFA [39]; for CD196 and CD196 *prdB*::CT), TCCFA supplemented with 50 µg/mL rifampicin and 20 µg/mL erythromycin (for CD2015), or TCCFA supplemented with 25 µg/mL kanamycin (for R20291, R20291 Δ*grdA*, and R20291 Δ*ldhA*). Samples were centrifuged at ~3,000 × *g* for 5 min to pellet cells. Supernatants were removed, and pellets and supernatants were stored at <−20°C.

### Microbial community analysis by 16S rRNA gene sequencing

DNA was extracted from cell pellets using the BioSprint 96 One-For-All Vet processing kit (Qiagen) according to instructions with the following modifications. Prior to extraction, cells were resuspended in Buffer ASL (Qiagen) and added to sterile deep well plates (Axygen) containing 0.1 mm Zirconia beads (BioSpec Products). Cells were disrupted by bead beating for 2 minutes at 1,800 rpm on a FastPrep 96 homogenizer (MP Biomedicals). DNA was amplified in duplicate with Phusion polymerase using barcoded primers 515F and 806R that target the 16S rRNA gene as previously described (41, 44), then sequenced on an Illumina MiSeq using 2 × 250 kits according to the manufacturer’s protocol. All sample processing and sequencing were performed by the investigators at the University of Nebraska-Lincoln using equipment shared by members of the Nebraska Food for Health Center. Fastqs were processed by mothur 1.41.3, removing chimeras identified by uchime, mapping sequences against Silva release 132, and clustering OTUs at 99% identity using the OptiClust algorithm (66–68). Mothur 1.48.1 was used to rarefy samples to 6944 sequences and to calculate alpha (observed OTUs, Inverse Simpson) and beta diversity (Jaccard and Bray-Curtis dissimilarity) metrics on rarefied data. Code for sequence processing can be found in File S3; Excel worksheets for processing of beta diversity data can be found in File S4. An OTU table of rarefied data can be found in Table S2, and compiled data from sequencing analysis can be found in Table S3.

### Bile salt measurements by liquid chromatography-tandem mass spectrometry

Quantitative analysis of the bile salt levels contained in conditioned-BRM3 culture media sample supernatants was performed by the Texas Children’s Microbiome Center Metabolomics and Proteomics Mass Spectrometry Laboratory (TCMC-MPMSL) using previously published methods (69, 70). Briefly, conditioned-BRM3 growth media samples were removed from the fecal communities cultured in the individual bioreactors using sterile needles and syringes. Samples were added into sterile tubes, and the bacterial cells were pelleted by centrifugation at 3,000 × *g* for 5 min. Clarified supernatants were then sterile filtered using 96-well 0.2 µM polyvinyl difluoride (PVDF) filter plates by centrifugation at 200 × *g* for 5 min. The 96-well, deep well plates used to capture sample filtrates were covered with silicone cap mats and the capped samples were wrapped in parafilm to ensure the fixture of the capmats during shipment. The cell-free conditioned media samples were stored frozen at −80°C pending shipment to the TCMC-MPMSL. The cell-free conditioned media samples were shipped frozen on dry ice, and upon receipt by the TCMC-MPMSL, were stored at −80°C until analysis.

All cell-free conditioned media samples were thawed at ambient temperature on a laboratory benchtop. Once thawed, the entire sample volume was transferred into individual 0.6 or 1.5 mL Eppendorf tubes, and all samples were vortex-mixed using a multi-tube vortexer. Preliminary work indicated that 10-fold and 1,000-fold dilutions were suitable to measure the taurocholic acid (TCA) content, and the cholic acid (CA) and deoxycholic acid (DCA) content, respectively, in each of the cell-free conditioned media samples using a common linear dynamic range of 0.977–1,000 ng/mL for CA, DCA, and TCA—the two different dilution procedures for each metabolite were described previously (70). These sample dilution steps were performed using a working internal standard (WIS) solution prepared in 1:1 methanol:water that contained 250 ng/mL each of D4-CA and D4-DCA as described (70). A 5 µL volume of sample was injected onto the SCIEX QTRAP 6500-based liquid chromatography-tandem mass spectrometry (LC-MS/MS) system, and bile acid concentrations in the conditioned media samples were back-calculated using the regression parameters of the standard curve as described previously (70). Sample data were filtered to remove any with total concentrations of cholate, deoxycholate, and taurocholate that were less than 1 μg/mL. The median level of total bile salts measured was 246 μg/mL; filtering removed 18 of 336 samples. All bile salt data, including the 18 samples that were filtered, can be found in Table S4.

While the levels of TCA, CA, and DCA in fresh bioreactor medium were measured at the time cultured samples were tested (69, 70), concentrations were not reported and were lost to follow-up. Therefore, we performed a broad screen of the bile acids/salt content of two independently prepared fresh preparations of BRM3-based bioreactor medium (with bovine bile) as well as a batch of BRM3 prepared without bile using another previously reported targeted metabolomics method that is capable of quantitatively measuring 16 bile acids/salts (71). This expanded method can quantify the bile acid/salt content of microbial media samples: CA, glycocholic acid (GCA), TCA, beta-muricholic acid (β-MCA), DCA, glycodeoxycholic acid (GDCA), taurodeoxycholic acid (TDCA), chenodeoxycholic acid (CDCA), glycochenodeoxycholic acid (GCDCA), taurochenodeoxycholic acid (TCDCA), ursodeoxycholic acid (UDCA), glycoursodeoxycholic acid (GUDCA), tauroursodeoxycholic acid (TUDCA), lithocholic acid (LCA), glycolithocholic acid (GLCA), and taurolithocholic acid (TLCA). Briefly, samples were prepared by diluting a 10 µL volume of a thawed cell-free, conditioned BRM3 media sample in a 90 µL volume of a WIS solution that contains deuterated analogs of each of the analytes listed above at concentrations of 250 nM for each prepared in 1:1 methanol:water (71). The samples were prepared directly in glass autosampler vials and the samples were vortex-mixed briefly prior to injecting a 10 µL volume onto the SCIEX QTRAP 6500-based LC-MS/MS system. Bile acid/salt concentrations in the fresh BRM3 medium samples were back-calculated using the linear regression parameters of the standard curve as described previously (71). All samples were tested in triplicate. Bile salt data for media can be found in Table S5. These studies confirmed the absence of major forms of bile acids/salts in BRM3 medium made without bile (Table S5).

### Data visualization and statistical analysis

The implementation of the LEfSe (47) algorithm in mothur 1.48.1 was used to identify potential OTUs correlated with *C. difficile* colonization from rarefied data as described in File S3. Abundance data for OTUs correlated with *C. difficile* colonization were manually curated from OTU abundance tables in Excel and visualized in GraphPad Prism version 10.2.3. R studio version 2022.07.0+548 running R version 4.2.3 was used to generate the heatmap in Fig. S2. Code and dependent software versions used for data analysis can be found in File S3. The remaining statistical analyses and visualization were performed using GraphPad Prism. Unless otherwise noted (Fig. 5; Fig. S8), the significance of differences at a single time point between two treatment groups was determined with a Mann-Whitney test and between more than two groups was determined with Kruskal-Wallis testing with Dunn’s correction for multiple comparisons. In Fig. 5 and Fig. S8, parametric statistics were used to determine significance due to the low number of replicates in one or more samples tested. One-way ANOVA with Brown-Forsythe and Welch correction for unequal variances and Dunnett T3 correction for multiple comparisons was used in Fig. 5, whereas a two-tailed Student’s *t*-test with Welch’s correction for unequal variances was used in Fig. S8. For analysis of repeated measures shown in Fig. 1C and D and Fig. S1E and F, a mixed-effects model was used to infer statistical significance with time and reactor as the fixed effects and treatment as the random effect. For CFU/mL data, values below the limit of detection of the assay (333 CFU/mL) were reported as “300” to facilitate plotting.

## Data Availability

16S rRNA gene data have been deposited in NCBI’s Sequence Read Archive (SRA) under BioProject ID PRJNA729569. Sequence data from *grdA* and *ldhA* were deposited to NCBI SRA with accession numbers SRR31632037 and SRR31632038.

## SUPPLEMENTAL MATERIAL

File S1 msphere.01049-24-s0001.pdf Figures S1 to S8.

File S2 msphere.01049-24-s0002.xlsx Tables S1 to S5.

File S3 msphere.01049-24-s0003.txt Code for microbiota analysis.

File S4 msphere.01049-24-s0004.xlsx Worksheet for analysis of beta diversity.

File S5 msphere.01049-24-s0005.xlsx Tables S6 to S8, for use with code in File S3.

ASM does not own the copyrights to Supplemental Material that may be linked to, or accessed through, an article. The authors have granted ASM a non-exclusive, world-wide license to publish the Supplemental Material files. Please contact the corresponding author directly for reuse.
